# Perceptions and protective experiences regarding reproductive hazards among operating room healthcare workers of childbearing age: a qualitative study

**DOI:** 10.3389/fpubh.2026.1877652

**Published:** 2026-07-31

**Authors:** Min Jiang, Yu Wang, Zhen Song, Dandan Shan, Hui Zhi, Qingwei Zhang, Aobo Liu, Qian Zhang, Wenjing Xu, Ke Fan

**Affiliations:** 1 Department of Anesthesiology, Henan Provincial People's Hospital, Zhengzhou, Henan, China; 2 Department of Nursing, Fuwai Central China Cardiovascular Hospital, Zhengzhou, Henan, China; 3 Department of Plastic Surgery, Henan Provincial People's Hospital, Zhengzhou, Henan, China

**Keywords:** healthcare workers of childbearing age, protective behaviors, reproductive hazards, risk perception, surgical smoke

## Abstract

**Introduction:**

Occupational reproductive hazards have a significant negative impact on the physical and mental health of healthcare workers, leading to risks of infertility and adverse pregnancy outcomes that far exceed those of the general population. However, the protective dilemmas and lived cognitive experiences regarding reproductive hazards among multidisciplinary teams of childbearing age within the complex, high-pressure environment of the operating room remain largely neglected in the fields of occupational medicine and nursing management.

**Methods:**

This study aimed to explore the perceptions of reproductive hazards and the experiences of adopting preventive measures among operating room healthcare workers of childbearing age. Using purposive sampling, face-to-face interviews were conducted with eligible participants. Data were organized and analyzed utilizing Colaizzi's seven-step phenomenological method.

**Results:**

The qualitative analysis revealed three main themes: (1) inconsistency between knowledge and practice regarding reproductive protection; (2) barriers to adopting protective measures; and (3) the conflict between a culture of dedication and individual health responsibility.

**Conclusions:**

This study provides new insights into the occupational reproductive protection dilemmas faced by operating room healthcare workers of childbearing age. It highlights the necessity for comprehensive interventions targeting issues such as the collective neglect of male reproductive risks, insufficient resource allocation, and team-based protection dilemmas reliant on interpersonal negotiation. Healthcare policymakers and managers should provide greater organizational support and foster a non-punitive safety culture, empowering healthcare workers to effectively safeguard their occupational reproductive health rights.

## 1 Introduction

As the core area of a hospital, the operating room (OR) is associated with various occupational exposure risks. Among these, reproductive issues induced by occupational exposure have emerged as a major challenge in global occupational health. Epidemiological data demonstrate that the reproductive health status of OR healthcare workers is significantly poorer than that of the general population. For instance, the incidence of pregnancy complications—such as threatened abortion, premature birth, fetal growth restriction, and congenital malformations—among female surgeons in the United States reaches 35.3%, compared to only 14.5% in the general population (1). Their infertility rate is approximately 32.0%, far exceeding the 10.9% observed in the general population, with 76% of those affected relying on assisted reproductive technology to conceive (2, 3). The risk of infertility is particularly prominent in specialties such as otolaryngology, general surgery, and orthopedics (2). In China, relevant epidemiological data reveal a similarly severe reproductive health crisis among this demographic: the spontaneous abortion rate and the incidence of congenital malformations in offspring among OR nursing staff are 1.3 to 2 times and 1.6 times higher, respectively, than those of non-occupationally exposed populations (4, 5). Furthermore, the overall spontaneous abortion rate among married female healthcare workers is as high as 14.2% (6). Reproductive hazards not only directly threaten the physical and mental health of healthcare professionals but also exacerbate staff turnover and occupational burnout, profoundly impacting the overall efficiency and safety culture of the healthcare system (3, 7).

Reproductive hazards refer to substances, environments, or activities that can adversely affect the reproductive health of men and women, or the ability of couples to produce healthy offspring. These hazards encompass chemical, physical, and biological factors, exerting negative impacts across the entire life cycle, including sexual function, fertility, pregnancy, childbirth, and lactation (8). OR healthcare workers are chronically exposed to complex physical, chemical, and biological reproductive risk factors. First, waste anesthetic gases (WAGs) can leak from masks or be exhaled by patients, lingering in the ambient air. Chronic low-dose exposure to WAGs can trigger endocrine disruption and genotoxicity in germ cells (9, 10). Second, surgical smoke generated by electrosurgical equipment contains benzene, formaldehyde, and ultrafine particles, which significantly increase the risk of adverse pregnancy outcomes (11). Cumulative exposure to high-level disinfectants and ionizing radiation has been proven to be closely related to declining fertility and fetal malformations (12, 13). Additionally, circadian rhythm disruption caused by shift work, prolonged standing, and heavy workloads further impairs reproductive endocrine function (14, 15).

Although international occupational safety and health organizations, such as the U.S. National Institute for Occupational Safety and Health (NIOSH), the Occupational Safety and Health Administration (OSHA), and relevant professional societies have issued exposure limits and protective guidance for specific hazards (16–20), healthcare workers of childbearing age still demonstrate low adherence to protective guidelines in clinical practice. On the one hand, protective equipment has inherent limitations. For example, over 55% of healthcare workers report that existing lead aprons fail to provide effective protection (21); the lack of maternity-designed lead aprons imposes a heavy physical burden and causes musculoskeletal pain (22). On the other hand, fearing marginalization, compromised career promotion, or increased workload for their colleagues, many female healthcare workers choose to conceal their preconception or pregnancy status, thereby foregoing the opportunity to request a transfer away from high-risk exposure environments (23).

At the institutional level, although general occupational health departments and generic pregnancy-accommodation protocols exist, these frameworks lack micro-environmental specificity for the perioperative domain. Standard policies fail to account for the multidisciplinary, interdependent, and time-critical nature of surgical workflows, ultimately delegating protective behaviors to individual compliance rather than systemic organizational support (24). Crucially, the OR maintains a rigidly hierarchical interpersonal matrix; frontline nurses and junior trainees lack the institutional discourse power to disrupt a surgeon's workflow or initiate environmental safety interventions. Because surgical interventions prioritize uninterrupted clinical execution, mid-procedure disengagement for self-protection is functionally precluded, rendering reproductive protection in the OR uniquely complex and distinct from conventional outpatient or general ward environments.

While the existing body of literature provides extensive quantitative insights into the toxicological mechanisms of specific hazards or descriptive statistics on PPE adherence (25, 26), qualitative and theoretical explorations remain restricted to macro-ethical or generic administrative models. There is a profound scarcity of empirical data capturing the authentic, lived cognitive experiences and behavioral dilemmas of multidisciplinary OR teams navigating these reproductive threats (27). Given that safeguarding reproductive health is critical to the sustainability of the healthcare workforce and the consolidation of institutional safety, an in-depth qualitative inquiry is urgently required (28, 29).

Shifting the analytical lens from individual knowledge deficits to the systemic, organizational, and cultural mechanisms that constrain protection—specifically, the gendered neglect of male reproductive risks, critical resource shortfalls, and vulnerabilities in team-based collaboration. This study utilizes a descriptive qualitative design to explore the risk perceptions and lived protective experiences regarding occupational reproductive hazards among OR healthcare workers of childbearing age. Ultimately, this investigation aims to provide an empirical and theoretical foundation for transitioning from an individually oriented compliance paradigm toward a robust organizational support framework.

## 2 The study

This study aimed to explore the perceptions of OR healthcare workers of childbearing age regarding reproductive hazards, as well as their lived experiences when adopting preventive measures. The specific research questions included: (1) How do participants perceive the reproductive risk factors present in the OR? (2) What protective measures do participants implement in their actual clinical practice? (3) What barriers or resistance are encountered during the implementation of protective measures, and how are these challenges navigated? (4) What systemic support is required from national policymakers and hospital administration?

## 3 Methods

### 3.1 Study design

This study employed a descriptive qualitative design (30) anchored in the constructivist paradigm (31) to explore the perceptions and protective experiences regarding occupational reproductive hazards among operating room healthcare workers of childbearing age. This epistemological stance posits that risk perceptions and protective behaviors are multidimensionally constructed phenomena generated through interactions within specific sociocultural contexts. Specifically, these constructs are profoundly shaped by individual clinical cognitive capacities, internal power dynamics within multidisciplinary teams, and the institutional culture of occupational dedication. By bracketing the researchers‘ *a priori* assumptions, this approach authentically captured participants' objective encounters and subjective lived experiences while averting excessive theoretical deduction. To achieve the research objectives, a pilot-tested semi-structured interview guide was utilized to encourage in-depth exploration (32). The manuscript was prepared in accordance with the Standards for Reporting Qualitative Research (SRQR) (33).

### 3.2 Participant recruitment

This study was conducted at a large tertiary hospital in China that provides comprehensive surgical services. Recruitment invitations were initiated by M.J. and Y.W. Eligible healthcare workers were included in this study; the inclusion criteria are detailed in Table 1. This study employed a purposive sampling strategy. A total of 31 eligible individuals were invited, and all agreed to participate (zero refusals). The sample size was guided by the concept of “information power” and data saturation. Sufficient and relevant data were collected to align with the research objectives.

**Table 1:** Recruitment criteria for operating room healthcare workers of childbearing age.

Inclusion criteria
•**Age:** Healthcare workers aged 18 to 45 years.
•**Professional qualifications:** Licensed surgeons, anesthesiologists, operating room (OR) nurses, and nurse anesthetists.
•**Informed consent:** Fully informed of the study's purpose and voluntarily agreeing to participate.

### 3.3 Data collection

To obtain detailed insights into participants' perspectives on OR reproductive hazards, a semi-structured interview guide was developed based on an extensive literature review and expert consultation. The initial guide was pilot-tested with 10 participants representing different OR roles, and subsequently refined based on their feedback and expert recommendations (32). Data from these 10 pilot interviews were excluded from the final analysis. The finalized semi-structured interview guide is presented in Table 2.

**Table 2:** Semi-structured interview guide.

Semi-structured interview guide
•What is your understanding of reproductive hazards?
•What factors in the OR do you believe contribute to reproductive hazards?
•How do you typically protect yourself from these reproductive hazards?
•What challenges or situations do you encounter when adopting or promoting protective
•Measures against reproductive hazards?
•How do you overcome the difficulties or resistance encountered during protection?

Between July 18, 2025, and October 13, 2025, researchers conducted face-to-face, semi-structured interviews in a quiet, private setting. Prior to each interview, written informed consent was obtained, confirming that participants understood the study details, agreed to participate, and consented to audio recording. Following each interview, the interviewer carefully reviewed the transcripts and requested participants to clarify, add to, or modify any questionable areas (participant checking) (34). Audio recordings were transcribed verbatim using transcription software and cross-checked against the original audio to ensure authenticity (35).

### 3.4 Data analysis

Data collection and analysis were conducted concurrently in an iterative manner. Within 24 h of completing each interview, audio recordings were transcribed verbatim and imported into NVivo 14 software to facilitate data management and coding. The analysis strictly adhered to Colaizzi's seven-step phenomenological method via an inductive approach: (1) reading transcripts repeatedly to achieve data immersion; (2) independently extracting significant statements directly pertinent to the investigated phenomenon by two blinded researchers; (3) formulating deeper meanings from these statements; (4) clustering similar formulated meanings into subthemes and themes; (5) developing an exhaustive description of the subjective lived experiences; (6) generating the fundamental structure of the phenomenon; and (7) verifying the authenticity of the findings with participants through member checking. Any coding discrepancies were resolved via expert consensus meetings. The final themes were named and defined through collaborative team discussions, culminating in the integration and refinement of subthemes into three overarching core themes (36). This iterative process was repeated for each subsequent interview, facilitating continuous data collection and analysis. The coders observed that no new coding categories or themes emerged after the 23rd interview; to rigorously confirm data saturation, recruitment and interviewing were extended. After the 31st interview confirmed that no new information was generated, the team unanimously agreed to cease data collection (37, 38).

### 3.5 Ethical considerations

This study was approved by the Institutional Review Board of Henan Provincial People's Hospital (Approval No. 165, 2025). Prior to each interview, all participants were provided with a detailed explanation of the study's purpose, procedures, potential risks, and benefits, after which written informed consent was obtained. Participants were informed of their right to ask questions or withdraw from the study at any time without facing any adverse consequences. To protect participants' privacy, all identifiable information was anonymized using numerical codes and securely stored in a password-protected folder.

### 3.6 Rigor and reflexivity

The methodological rigor of this study was strictly maintained by adhering to Lincoln and Guba's (39) four criteria for trustworthiness: credibility, dependability, confirmability, and transferability. The research team comprises nurses and surgeons with clinical experience in the OR. Additionally, an expert proficient in qualitative methodologies (W.Y.) trained all team members prior to the study's commencement to minimize interviewer bias (40). To mitigate potential cognitive interference stemming from this experiential background, the two researchers responsible for data collection and analysis engaged in reflexivity discussions with the corresponding author prior to data collection to bracket their *a priori* assumptions regarding OR reproductive hazards. Credibility was established through peer debriefing—wherein another researcher (J.M.) documented and verified the findings following each interview (39) —and member checking, which involved returning transcripts and preliminary analytical findings to participants for clarification and confirmation. Dependability and confirmability were safeguarded by establishing an exhaustive audit trail, continuously maintaining reflexive journals (40), and employing investigator triangulation; two independent coders (J.M. and W.Y.) cross-checked the data (41); and any discrepancies were resolved by an expert panel. Transferability was supported by providing thick descriptions of the participants' demographic characteristics and the specific research context.

## 4 Results

### 4.1 Participant characteristics

All 31 eligible participants agreed to participate, were included in the study, and completed the interviews. To capture the diverse perspectives within the multidisciplinary team, the cohort was explicitly categorized into nursing staff (*n* = 25) and physicians (including surgeons and anesthesiologists, *n* = 9). The mean age was 33.9 years (SD = 5.7, range 22–44 years). The mean clinical work experience was 10.3 years (SD = 7.2, range 1–25 years). Regarding professional titles, 12 participants held senior titles and 19 held intermediate titles. Furthermore, 67.7% of the participants possessed a graduate degree (Table 3).

**Table 3:** Demographic characteristics of the participants (*n* = 31).

Participant characteristics	*n* (%)
Age (years)	
18–25	2 (6.45)
26–30	9 (29.03)
31–35	7 (22.58)
36–40	8 (25.81)
41–45	5 (16.13)
Profession	
Nurses	14 (45.16)
Physicians	17 (54.84)
Professional title	
Associate chief nurse/physician	11 (35.50)
Nurse-in-charge/attending physician	17 (54.80)
Nurse/resident physician	2 (6.50)
Managerial role	
Yes	5 (16.10)
No	26 (83.90)
Clinical work experience (years)	
1–5	10 (32.26)
6–10	8 (25.81)
11–20	10 (32.26)
≥ 21	3 (9.68)

### 4.2 Main findings

**Figure 1:**
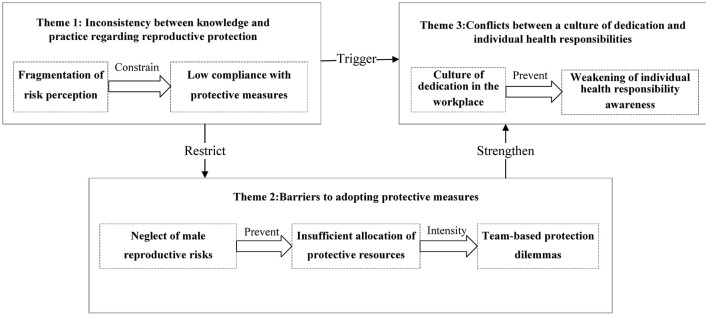
Framework of reproductive hazards among operating room healthcare professionals of childbearing age.

Through in-depth interviews with 31 operating room healthcare workers of childbearing age, three core themes and seven subthemes were extracted (Table 4). As systematically illustrated in Figure 1, the interrelationships among these themes constitute a causal and progressive theoretical framework driving low compliance with reproductive protection. The ‘culture of dedication' (Theme 3) operates as the fundamental trigger, consistently subordinating individual health needs to occupational responsibilities and provoking a profound conflict with personal health awareness. This cultural context subsequently exacerbates systemic flaws, encompassing the collective neglect of male reproductive risks, insufficient resource allocation, and hierarchical team-based protection dilemmas reliant on interpersonal negotiation (Theme 2). Consequently, these overwhelming systemic barriers severely constrain individual agency, ultimately manifesting as the ‘inconsistency between knowledge and practice' (Theme 1). This indicates that low compliance does not merely stem from fragmented risk perceptions, but rather from a coercive environment where healthcare professionals are forced to abandon protective behaviors despite recognizing the risks. To mitigate these hazards, healthcare workers employ individualized and contextualized coping strategies, attempting to strike a balance between maintaining high-intensity workloads and practicing self-reproductive protection (Figure 1).

**Table 4:** Extraction and analysis of core meanings.

Theme	Subtheme	Core codes
1. Inconsistency between knowledge and practice regarding reproductive protection	1.1 Fragmentation of risk perception	Relying on vague somatic perception due to cognitive ambiguity; experiencing uncertainty and psychological anxiety regarding exposure thresholds.
	1.2 Low compliance with protective measures	Prioritizing surgical cooperation absolutely over personal safety; deliberately or passively omitting protections due to time constraints, urgency, and severe physical/mental exhaustion.
2. Barriers to adopting protective measures	2.1 Neglect of male reproductive risks	Erroneously framing reproductive health exclusively as a female responsibility; trivializing male high-risk exposures and demonstrating gender disparities in resource allocation.
	2.2 Insufficient allocation of protective resources	Lacking essential safety barriers due to severe shortages and lagging investments in protective facilities; experiencing psychological imbalance and professional burnout.
	2.3 Team-based protection dilemmas	Lacking formal enforcement authority to mandate safety protocols; relying on non-punitive interpersonal negotiations (e.g., humor, pleading) and compensatory assistance due to the absence of institutional safeguards.
3. Conflict between a culture of dedication and individual health responsibility	3.1 Culture of dedication in the workplace	Internalizing the “endurance and dedication” professional norms under high-intensity operations; employing self-rationalization driven by a conformity mentality to maintain the status quo.
	3.2 Weakening of individual health responsibility awareness	Demonstrating unconditional dedication by normalizing and passively accepting reproductive risks; suffering from continuous psychological anxiety and role-related guilt.

#### 4.2.1 Theme 1: inconsistency between knowledge and practice regarding reproductive protection

This theme revealed the discrepancy between cognition and behavior regarding occupational reproductive protection among OR healthcare workers. Due to a lack of systematic knowledge regarding reproductive protection, participants exhibited fragmented perceptions of occupational exposure risk factors, causing their risk assessments to rely heavily on vague perceptions of somatic discomfort. Concurrently, within a highly efficient and fast-paced work environment, participants' willingness to protect themselves often compromised with clinical tasks. This led to a significant decline in protective compliance, presenting a severe dilemma of inconsistency between knowledge and practice.

##### 4.2.1.1 Subtheme 1.1: fragmentation of risk perception

Although participants could identify certain risks, the lack of clear safety standards made it difficult for them to consistently implement self-protection in their daily work. Regarding radiation, Participant 23 stated, “I know radiation is harmful and I need to wear a lead apron, but I have no idea what dose is considered safe or how much impact a single exposure has.” Similarly, Participant 15 observed, “For surgical smoke, especially from high-efficiency equipment like ultrasonic scalpels, the crushed and feathered particles are smaller, and the smaller the particles, the greater the hazard. Does the surgical smoke generated by electrocautery have less impact?” In addition to doubts about perceived risks, psychological unease stemmed more from physical sensations. Participant 13 described, “Disinfectants, meals, mood swings, staying up late, radiation, and narcotics can all affect reproductive health. Staying in the OR for a day makes me feel unwell when I come out; it's not as comfortable as staying in the general ward for a day. I just feel tired.” Participant 22 added, “Living in the OR environment for a long time, I can't articulate exactly what is bad, but thinking carefully, the leakage of ether during anesthesia induction, the radiation from iodine seeds, and the constant bending over during patient positioning are all bad for us.”

##### 4.2.1.2 Subtheme 1.2: low compliance with protective measures

In this study, under high-efficiency and high-intensity workloads, participants frequently abandoned protective equipment and measures, either actively or passively. Consequently, their willingness to protect themselves could not be translated into effective interventions. The experience of Participant 25 revealed this frustration: “The equipped smoke evacuation electrocautery pencils are hard to use. The surgeons complain that they affect suction, so the smoke evacuation tubing is often cut off.” Participant 8 articulated this phenomenon clearly: “I usually don't ignore the behavior of not taking protective measures, but first, you have to cooperate with the surgery.” Cooperating with the surgery was placed in an indisputable priority position, becoming the prerequisite for evaluating all personal behaviors (including whether to persist with protection). Participant 9 also described this dilemma: “You don't have that much time to adopt protective measures. It gets quite busy at the beginning of the surgery, leaving no time to wear a lead apron. Many people just say ‘forget it'.” Long-term physical and mental exhaustion further weakened the motivation to translate limited cognition into sustained protective behaviors. Participant 6 confessed: “Our labor intensity is so high that I don't want to do any work other than caring for the patients.”

#### 4.2.2 Theme 2: barriers to adopting protective measures

This theme revealed four factors making protective measures difficult to implement: conceptually, reproductive risks were often erroneously deemed a female responsibility; materially, hospitals severely underinvested in resources such as protective equipment; collaboratively, nurses lacked the authority to enforce safety protocols within multidisciplinary surgical teams; and individually, participants developed a range of personalized, context-dependent coping strategies. These difficulties hindered healthcare workers' willingness to protect themselves from translating into effective clinical protective behaviors.

##### 4.2.2.1 Subtheme 2.1: neglect of male reproductive risks

In clinical practice, discussions, concerns, and responsibilities regarding reproductive health risks were often unconsciously and exclusively tied to females, resulting in a prolonged neglect of the reproductive risks faced by male healthcare workers. Participant 2 explicitly pointed out: “When discussing this [reproductive hazards] in the department, it seems to default to female colleagues, especially those planning to have children.” Based on this organizational default, resource allocation for protective strategies exhibited significant gender disparities, as described by Participant 1: “For females with reproductive needs, we try to keep them away from operating rooms with hazardous chemicals and radiation..., and we give them certain accommodations in scheduling... For males with reproductive needs, it is just about quitting smoking and drinking, and eating regularly.” Furthermore, in team collaborations, placing males in high-hazard environments was even viewed as a reasonable resource allocation strategy. As Participant 5 noted: “They always think it might be more reasonable for girls to enter less and boys to enter more into the radiation surgical areas.” Male practitioners also generally displayed an avoidant attitude and low risk perception regarding reproductive hazards. Manager P1 confessed: “For males, others are not willing to talk to you about reproductive issues either.” Compared to the high vigilance of females, males‘ attitudes toward protection often appeared flippant and perfunctory. Participant 4 pointed out: “If it were a female, she might care more, but a male might just...” More severely, males' neglect of occupational reproductive risks deepened after fulfilling their personal family planning goals. Participant 15 stated bluntly: “I already have two children, why should I still consider these things?”

##### 4.2.2.2 Subtheme 2.2: insufficient allocation of protective resources

Healthcare institutions severely underinvested in safety and protective facilities. The shortage and aging of critical equipment, such as dedicated smoke evacuation systems, biological safety cabinets, and environmental monitors, deprived healthcare workers of basic protective barriers. Regarding surgical smoke exposure, Participant 7 stated: “Currently, there is a lack of comprehensive smoke evacuation devices.” In addition to physical hazards, safety defenses against high-risk chemical exposure also had significant blind spots. Participant 8 indicated: “We don't have a dedicated cabinet for mixing chemotherapy drugs. Everyone just wears gloves and mixes drugs directly; the [drug aerosol] falls onto the skin.” Facing the urgent need for safety supplies, managers also appeared powerless. Participant 12 confessed to the resistance encountered when requisitioning protective facilities: “The hardware we need is not provided. For instance, the department has applied for waste gas scavenging systems multiple times, but due to various reasons, they were never implemented in the end.” This predicament not only restricted protective behaviors but also triggered psychological imbalance and burnout among healthcare workers. Participant 5 explained this profound negative effect: “The severe inadequacy of hardware equipment is a fatal flaw. Even if my protective awareness has increased and my willingness has strengthened, the outdated facilities not only fail to provide protection but actually create a psychological gap.”

##### 4.2.2.3 Subtheme 2.3: team-based protection dilemmas

In multidisciplinary surgical teams, the effective implementation of reproductive protection measures relied heavily on communication and negotiation among team members, rather than institutionalized safety protocols. Because the surgical team's core objective was completing the surgery, healthcare workers were often forced to compromise when facing conflicts between protective needs and clinical tasks. As Participant 1 analyzed: “At work, you are not the one taking the lead... to successfully complete the task, sometimes you have to abandon some peripheral things.” To maintain the efficiency of multidisciplinary collaboration, frontline healthcare workers adopted strategies such as negotiating or actively providing protective tools and assisting in executing protective measures. Participant 25 clearly articulated this phenomenon: “I usually tell the doctors to take protective measures in a humorous way; anyway, I can't force them to do it.” Participant 9 demonstrated assisting in executing protective measures: “When doctors don't take protective measures, if I can manage, I will actively use the suction to remove the surgical smoke.” However, this collaborative model, which was highly reliant on individual consciousness, demonstrated significant vulnerability. When protective suggestions were not adopted, healthcare workers usually had no choice but to passively accept the situation. Consequently, many protective behaviors ended right after the reminder, lacking institutionalized safeguard mechanisms to ensure execution. Furthermore, distinct professional roles dictated different protective dilemmas. Physicians, driven by the imperative of surgical efficiency and technical execution, reported that protective measures (e.g., bulky lead aprons or cumbersome smoke evacuation tubing) frequently interfered with operative precision, leading to the active or passive abandonment of these measures. Conversely, nursing staff in supporting roles faced hierarchical barriers; they lacked the formal institutional authority to enforce safety protocols. When facing dominant figures, such as surgeons, who failed to adopt standardized protective measures, the lack of institutionalized psychological safety made it difficult for nurses to compel compliance.

#### 4.2.3 Theme 3: conflict between a culture of dedication and individual health responsibility

When the occupational vocation of saving lives potentially threatened personal health, healthcare workers endured not only physiological risks but also psychological anxiety and guilt. This was not merely an issue of work-life balance, but an internal conflict concerning self-worth, professional identity, and personal wellbeing.

##### 4.2.3.1 Subtheme 3.1: culture of dedication in the workplace

Behind the highly efficient operation of the OR lay the immense sacrifices healthcare workers made regarding their personal lives. Faced with this severely imbalanced rhythm, they unconsciously chose collective endurance. The description by Participant 4 was highly evocative: “The nature, intensity, and targets of OR work require us to have a high sense of responsibility. Every day at work, we are spinning non-stop like a top.” Participant 24 relayed the astonishment of a visitor: “Today I heard a visiting doctor say, 'If you don't get off work until seven or eight o'clock, is your family okay with that? Don't you tutor your children with their homework? Don't you feel anxious?”' The visitor's questions highlighted that the OR work schedule had deviated from the normal rhythm of social life. However, even when questioned externally, participants habitually used explanations like “Actually, our department takes good care of pregnant women” to balance the situation. This precisely demonstrated that while they recognized the problem, they were deeply entrenched in the contradiction of defending the status quo, further reinforcing the spirit of self-dedication. Participant 19 pointed out clearly: “All institutions promote an image of endurance and dedication for healthcare workers. Everyone is in this environment; if others don't complain, you feel embarrassed to speak up.” This conformity mentality, based on collective acquiescence, formed a powerful controlling force.

##### 4.2.3.2 Subtheme 3.2: weakening of individual health responsibility awareness

In a “patient-centered” clinical environment, healthcare workers possessed a “self-sacrificial mindset,” willing to exert extraordinary efforts at work and even sacrifice their own health for the benefit of the patients. Participant 1 clearly expressed this spirit of unconditional dedication: “During an on-call shift with orthopedic surgeries, you will face reproductive risks, but as a specialized orthopedic nurse, cooperating with such surgeries is your professional duty.” Participant 2 frankly admitted that hazards like surgical smoke were “impossible to prevent and can only be passively accepted,” helplessly confining risk control to the adjustment of personal lifestyle habits: “I can only start with myself.” To alleviate the psychological anxiety brought on by long-term high-risk exposure, some healthcare workers tended to use the prevalent societal fertility dilemmas to downplay occupational environmental risks. For instance, Participant 10 emphasized, “Individual differences are too great, and there is too much infertility nowadays.” Notably, even when healthcare workers had a full awareness of the hazards, it was difficult to translate this into actual protective actions. Although Participant 18 clearly recognized that occupational exposure “might affect pregnancy, fetal development, and the subsequent growth of the child,” the inability to change the status quo turned this awareness into a continuous psychological burden.

## 5 Discussion

This study explored the cognitive limitations and lived protective experiences of operating room healthcare workers of childbearing age when coping with occupational reproductive hazards. Based on participants' accounts, the findings suggested that although reproductive protection measures are widely regarded as crucial for safeguarding the health of healthcare workers and their offspring, protective behaviors frequently appeared to compromise with surgical efficiency under the perceived influence of high-intensity clinical workloads and a patient-centered culture of dedication. Participants described this conflict as having a severe impact on their physical and mental health. These interpretive propositions enrich the existing literature on occupational protection compliance, suggesting how medical safety culture and multidisciplinary interpersonal collaboration may disrupt the daily reproductive protection of healthcare workers, rather than attributing this failure solely to a lack of safety knowledge or a shortage of protective equipment.

Consistent with previous occupational exposure surveys and epidemiological studies, the empirical findings based on participants' accounts suggested that OR healthcare workers of childbearing age generally exhibited low compliance with reproductive protective measures (42, 43). However, previous studies have predominantly attributed protection failures to weak individual safety awareness or a lack of professional knowledge (43). The innovation of this study lies in transcending the simplistic “knowledge-behavior” linear causal model (44, 45) and interpreting the inconsistency between knowledge and practice as a passive compromise within a high-pressure clinical environment. These interpretive propositions suggest how cognitive limitations and a heavy-workload environment jointly hinder the execution of reproductive protective behaviors. Participants reported that when facing hazards lacking clear quantitative standards, such as waste anesthetic gases and surgical smoke, their risk assessments shifted toward relying on subjective somatic perceptions, such as physical fatigue. According to Karasek's “Job Demand-Control” model (46), the superimposition of highly efficient surgical demands and low self-protection control appears to trigger severe occupational stress. Driven by the clinical imperative where surgical cooperation takes absolute priority, heavy workloads are perceived to severely weaken fragmented safety cognition, potentially leading to the active or passive abandonment of protective behaviors (2, 47).

When exploring the barriers to adopting protective measures, participant accounts suggested that a severe shortage of protective equipment acted as a primary constraint on protective behaviors, which aligns with existing conclusions regarding insufficient occupational health investment in healthcare institutions. However, this study breaks through the traditional environmental toxicology perspective by proposing that the imbalanced power dynamics within multidisciplinary teams and organizational gender biases may act as core factors. Unlike existing research that focuses primarily on the risk of pregnancy complications among female surgeons (2, 22), these interpretive propositions suggest that organizational culture appears to frame reproductive risks as an exclusively female issue. This gendered perspective may not only neglects the hidden occupational exposure risks for male practitioners (48) but also potentially allow allows healthcare institutions to inadvertently shirk their statutory responsibility of implementing universal protection (43). Furthermore, within multidisciplinary team collaboration, participants reported that nurses in a supporting role lack the formal authority to enforce safety protocols (49, 50). When facing dominant figures, such as surgeons, who fail to adopt standardized protective measures, the lack of institutionalized psychological safety was perceived to makes it difficult for nurses to compel compliance (50). Consequently, safety protection standards were frequently relegated to interpersonal negotiations, relying on strategies like humor or pleading. This collaborative model, which is highly reliant on individual consciousness and power dynamics, highlights the potential vulnerability of team-based protection efficacy within a rigidly hierarchical clinical organizational environment (51).

Based on participants' accounts, this study also revealed that healthcare workers described feeling deeply entrenched in a moral conflict between their dual identities as professional healers and future parents. This is highly consistent with literature exploring occupational burnout and psychological exhaustion among surgeons and intensive care staff due to high-pressure work (52). However, the profundity of this study is that it does not merely view the culture of dedication as a trigger for occupational burnout (52), but proposes it as a deep-seated organizational constraint mechanism. Under this insidious cultural constraint, the expectation of absolute priority for patient lives is perceived to chronically suppresses individuals' rights to health (2). Facing what they perceive as unchangeable organizational flaws and high-intensity clinical work, healthcare workers reported being forced to internalize organizational risks as personal moral responsibilities, adopting individualized and contextualized resilient defense strategies to digest these risks internally (53). These interpretive propositions pose a critical reflection on the classic systemic resilience theory in the field of healthcare quality and safety: the over-reliance on healthcare workers' endurance, physical and mental exhaustion, and reproductive compromises may superficially maintain the efficient operation of surgeries, but potentially masks deep-seated workplace safety vulnerabilities and resource shortages (54). This appears to allow management to continually shelve occupational health reforms, ultimately contributing to a vicious cycle where occupational injuries are institutionally defaulted (52, 53).

### 5.1 Implications for policy and practice

These interpretive propositions yield crucial empirical insights for occupational health management and clinical practice. First, healthcare facilities must prioritize environmental safety interventions over individual personal protective equipment. This necessitates installing dedicated smoke-evacuation systems at all electrosurgical workstations, utilizing biological safety cabinets for cytotoxic drug preparation, and deploying real-time environmental monitors for waste anesthetic gases and particulates (25, 43). Second, a hospital-level reproductive health committee should establish a dynamic early-warning and job-reassignment mechanism. By confidentially identifying staff during the preconception or pregnancy stages for automatic reassignment away from high-hazard procedures, institutions can eliminate the dependence on self-disclosure and mitigate gender disparities in risk management (2, 48). Third, administrators should mandate the integration of reproductive protection protocols into the multidisciplinary surgical safety checklist as a formal “time-out,” thereby structurally empowering frontline nurses with the institutional authority to halt high-risk procedural steps until adequate protection is verified (49–51). Fourth, mandatory, de-gendered continuing medical education covering reproductive toxicology and occupational safety must be implemented to correct fragmented risk perceptions and alleviate the moral distress and psychological burdens experienced by healthcare workers balancing high-intensity clinical demands with personal health protection. Ultimately, these systemic organizational interventions facilitate a paradigm shift from individual-level compliance to robust, non-punitive institutional support.

### 5.2 Strengths and limitations

This study transcends the traditional focus on individual compliance in occupational health research. By innovatively anchoring the root cause of OR reproductive protection failure in the dual barriers of a culture of dedication and an imbalanced hierarchical structure, it achieves a theoretical elevation from individual attribution to systemic flaws. However, several limitations must be acknowledged. First, recruiting participants from a single large public tertiary hospital in China necessitates cautious interpretation regarding transferability. The identified “culture of dedication” and gendered reproductive responsibilities may be heavily influenced by the specific national context—characterized by collectivist professional norms and high surgical volumes—and institutional resource constraints. Consequently, these findings are primarily applicable to similar tertiary surgical settings and require contextual validation prior to extrapolation to primary care, outpatient clinics, or heterogeneous healthcare systems. Second, the cross-sectional qualitative design precludes longitudinal tracking of how risk perceptions and protective behaviors evolve across different career trajectories or reproductive life-cycle stages. This study did not systematically analyze specific confounding variables, such as cumulative prior night shift exposure and reproductive history; these potential moderating effects remain to be explored.

### 5.3 Recommendations for future research

Building upon the identified themes, future research should prioritize three key directions. First, longitudinal studies are warranted to map the dynamic evolution of risk perceptions and protective behaviors across different career trajectories and reproductive stages, explicitly assessing the potential moderating effects of confounders such as parity and cumulative night-shift exposure, thereby facilitating tailored professional education for distinct occupational groups. Second, researchers should develop and prospectively evaluate complex organizational interventions—integrating environmental safety interventions, checklist-embedded safety “time-outs,” and mandatory continuing education—to determine their long-term efficacy in enhancing clinical protective compliance and team psychological safety. Third, future investigations should employ mixed-methods designs that integrate qualitative experiential accounts with objective biomarkers to substantiate the causal exposure-outcome mechanisms associated with high-risk microenvironmental factors such as surgical smoke and waste anesthetic gases.

## 6 Conclusions

This study provides an in-depth revelation of the lived experiences of operating room healthcare workers of childbearing age facing occupational reproductive hazards, alongside the dilemma of inconsistency between knowledge and practice in protection. The findings indicated that frontline staff generally possessed fragmented perceptions of reproductive risks. Simultaneously, the collective neglect of male reproductive risks, the shortage of protective resources, and team-based protection reliant on interpersonal negotiation collectively constituted barriers to the implementation of protective measures. More importantly, the culture of dedication exacerbated role conflicts among healthcare workers. Under the pressure of maintaining high-intensity surgical operations, they were frequently forced to compromise by sacrificing their personal health. Therefore, administrators must not only increase investment in protective resources but also foster a de-gendered and non-punitive organizational safety culture.

## Data Availability

The original contributions presented in the study are included in the article/supplementary material, further inquiries can be directed to the corresponding author.
